# Prevalence and Risk Factors of Dementia Among Older People of Gelao Ethnicity in China: A National Cross‐Sectional Study

**DOI:** 10.1002/cns.70897

**Published:** 2026-05-11

**Authors:** Xiaoling Zhao, Dan Meng, Xiaoli Yuan, Yong Luo, Jinghuan Gan, Mei He, Yuhong Long, Yan Zhang, Xia Wu, Bin Zhao, Pan Cai

**Affiliations:** ^1^ The Third Affiliated Hospital of Zunyi Medical University (The First People's Hospital of Zunyi) Zunyi People's Republic of China; ^2^ School of Nursing Zunyi Medical University Zunyi People's Republic of China; ^3^ Department of Nursing The Affiliated Hospital of Zunyi Medical University Zunyi People's Republic of China; ^4^ Beijing Friendship Hospital Capital Medical University Beijing People's Republic of China; ^5^ Zunyi Red Granite City Hospital Zunyi People's Republic of China; ^6^ Guilin University Guilin People's Republic of China

**Keywords:** associated factors, cross‐sectional study, dementia, Gelao ethnic group, prevalence

## Abstract

**Aims:**

Approximately 96% of the Gelao population lives in Guizhou Province, predominantly in Wuchuan and Daozhen counties. This study explored the prevalence of dementia and the factors associated with it among the Gelao ethnic group in this area.

**Methods:**

A stratified random cluster sampling method was used to conduct a face‐to‐face questionnaire survey with Gelao individuals aged ≥ 65 years from August to October 2022. The survey collected data on demographics, medical history, lifestyle, and dietary habits. Participants were assessed using the Chinese Mini‐Mental Status Examination (CMMS) and the Activities of Daily Living (ADL) scale. Dementia diagnoses were made according to DSM‐IV‐TR criteria.

**Results:**

Among the 2712 participants, the prevalence of dementia was found to be 24.74% (95% CI: 23.1–26.4). Multivariable analysis indicated that factors such as not residing in a nursing home, maintaining good tooth brushing habits, engaging in physical activity, visiting the market, playing mahjong, having a high income, drinking oil tea, and consuming *Houttuynia cordata* were associated with lower odds of dementia (all *p* < 0.05).

**Conclusion:**

The prevalence of dementia among older Gelao adults is notably high, especially among women. These results underscore the urgent need for targeted awareness campaigns, screening initiatives, and intervention strategies aimed at alleviating the dementia burden within this ethnic minority population.

## Graphical abstract

This study reveals a 24.74% prevalence of dementia among elderly Gelao people in China, with women's rates more than double those of men and substantially exceeding the national average. We identify distinct sociocultural protective factors—including community engagement and traditional diets—alongside risk factors. These findings highlight the urgent need for healthcare providers to increase awareness and implement standardized care to reduce the disease burden in this high‐risk group.

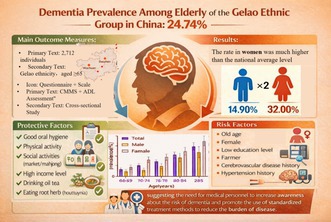

## 1 Introduction

As China's population ages, the number of patients with senile dementia has been continuously increasing yearly, imposing heavy socioeconomic and human resource burdens [1]. China is a large country with many ethnic groups, each with distinct cultural characteristics. Among the 56 ethnic groups in China, the Han ethnic group is the largest, accounting for more than 90% of China's population. They live in almost every region in China and own most property, technology, and resources [2, 3]. For the minority ethnic groups, populations face the challenge of limited health resources [4, 5]. In addition, considering factors such as government policies, regional economic development, and geographical conditions, which significantly affect the allocation of these health resources, the ethnic minority population who mainly live in remote regions and high‐altitude areas have high levels of underdevelopment compared to the Han population, which lives in lower‐altitude areas with better economies [6, 7, 8, 9]. In addition, studies have also shown that from the spatial distribution characteristics of the health level of the elderly population in China, people in the western regions (mostly ethnic minorities) of China, where the economy is less developed, experience low health levels [10, 11]. As a result, ethnic minorities have poorer health outcomes compared to non‐minorities [12, 13].

The Gelao ethnic group is an ancient ethnic group that has lived on the Yunnan‐Guizhou Plateau in southwest China for generations. About 96% of the Gelao population resides in Guizhou Province, mainly in the two counties, Wuchuan Gelao and Daozhen Gelao and Miao Autonomous counties [14]. Guizhou is located in the southwestern hinterland of China, exhibiting disparities in economic and cultural attributes compared to the developed areas in the central and eastern regions of China [15]. Most of the Gelao ethnic population live in remote rural areas of Guizhou. Throughout its extensive historical development, the ancestors of the Gelao ethnic group primarily resided in remote mountainous regions characterized by high altitudes, cold climates, and arid landscapes. Guizhou, situated in the southwestern interior of China, exhibits significant economic and cultural disparities when compared to the more developed central and eastern regions of China [15]. This topography and isolation resulted in limited interaction and exchange with the outside world [16]. Folk songs such as “On the Mountain Peak and at the Water's Edge,” “High Mountain Miao,” “Shuidong Family,” and “Gelao Lives in a Rocky Corner” authentically reflect the natural environment in which the Gelao people have lived for generations [17].

A survey found that the Gelao ethnic people in Guizhou are more likely to suffer from chronic diseases than the Han ethnic group [18]. The incidence rate of stroke among the Gelao ethnic people in Guizhou is as high as 76.36% for people in the 60–69 age group. It is significantly higher compared with that of the Han and other ethnic groups, which is ascribed to the unique dietary customs of the Gelao ethnic group [19]. Studies have shown that drinking *Camellia oleifera* Abel., eating roots of *Houttuynia cordata* Thunb., eating *Boletus edulis* Bull. ex Fr., and playing mahjong, etc. are the unique lifestyle and dietary habits of the Gelao ethnic people. However, current epidemiological research on elderly dementia in China has primarily focused on the Han elderly population, with no reports on the prevalence of dementia among the Gelao people.

Given the poor understanding of the prevalence and risk factors of elderly dementia in this minority group, this study not only fills the current gap in this area but also provides important reference data to guide the formulation of specific dementia management policies targeting the Gelao ethnic group. This will have a significant effect on the national health and long‐term development of the national economy.

## 2 Methods

### 2.1 Study Design and Setting

This population‐based cross‐sectional study was conducted from August to October 2022 in Daozhen and Wuchuan Gelao and Miao Autonomous Counties, Guizhou Province.

### 2.2 Sampling Method

A stratified cluster random sampling method with unequal ratios was implemented. Stratification was conducted based on geographical region, level of urbanization, and economic development status. In the initial stage, all 4 streets, 21 towns, and 4 townships within the two counties were classified into three strata: urban, suburban, and rural areas. In the subsequent stage, using a random number table, we selected two streets from the urban stratum (Duru Street and Yinzhen Street), two towns from the suburban stratum (Zhennan Town and Shangba Town), and two towns from the rural stratum (Zhuoshui Township and Old Town). In the final stage, 2–5 communities per street and 5–8 villages per township were randomly selected, with 1–2 additional households included as replacements for any non‐responses. All eligible residents in the selected communities and villages were invited to participate.

### 2.3 Research Subjects

The inclusion criteria for the study were as follows: (1) participants must be aged 65 years or older; (2) they must belong to the Gelao ethnic group; (3) they must be permanent residents of the surveyed area, defined as individuals with either a local registered residence or a residence period of 5 years or more, and must not have left the surveyed area during the study; (4) participants must have agreed to participate in the survey and signed an informed consent form.

The exclusion criteria included: (1) individuals with permanent household registration in the surveyed area who were away from the area for extended periods; (2) those diagnosed with severe schizophrenia; (3) individuals with significant impairments in hearing, vision, or language; (4) participants who were absent for three consecutive visits at different time points.

### 2.4 Sample Size Calculation

Based on a nationwide study, the prevalence of dementia among individuals aged 65 and older was approximately *p* = 9.11% [20]. To obtain an error not above 6% and a Z of 1.96 (*α* = 0.05), a sample of 386 (*n* = 386) was selected using the PASS software. As six sites were selected in this study (Duru Subdistrict, Yinzhen Subdistrict, Zhennan Town, Shangba Township, Zhuoshui Town, and Old Town), the total target sample size was 386 × 6 = 2316 participants.

### 2.5 Data Collection and Definitions

#### 2.5.1 General Information

The survey questionnaire collected data including demographic information (age, sex, education, and marital status), lifestyle factors (smoking history, alcohol intake history, consumption of *Camellia oleifera* Abel., consumption of *Houttuynia cordata roots*, and consumption of *Boletus edulis* Bull. ex Fr.), physical activity (visiting the market and playing mahjong), and past medical history (including cerebrovascular disease, headaches, diabetes, heart disease, and hypertension).

#### 2.5.2 Definitions of the Various Factors and Measurement Standards for the Related Indicators

According to Chinese standards, we defined body mass index (BMI) as ≥ 28 kg/m^2^ as obesity [21]. Smoking was considered as the consumption of at least one cigarette daily for over 1 year. In this context, one cigarette is approximately equivalent to 1 g of tobacco leaves. Alcohol consumption was characterized by the intake of more than 50 mL at least once a week for a duration exceeding 6 months. Physical activity was identified as participating in self‐reported voluntary exercise—including farm work, square dancing, walking, or running—for a minimum of 30 min per week. Hypertension was identified as a documented history of high blood pressure confirmed by diagnosis at a second‐class or higher hospital, or as having high blood pressure while receiving medication. Diabetes was defined as a confirmed diagnosis of diabetes at a second‐class or higher hospital, or as a patient receiving medication for diabetes. Cerebrovascular disease was characterized by a documented history of stroke with a clear diagnosis made at a second‐class hospital or higher. Additionally, conditions such as headache, heart disease, carbon monoxide poisoning, traumatic brain injury, and TIA were identified based on a history of these conditions, all of which required diagnosis at a second‐level hospital or above.

### 2.6 Assessment and Diagnostic Procedures

#### 2.6.1 Composition of Investigators

The investigation team comprised 15 members, including three experienced cognitive impairment specialists (two deputy chief physicians and 1 attending physician) and 12 graduate students majoring in cognitive impairment. All investigators were proficient in Guizhou local dialect and Putonghua. They were uniformly trained at the Third Affiliated Hospital of Zunyi Medical University before the investigation, and later retrained every 4 weeks during the study.

#### 2.6.2 Investigation and Screening Methods

The investigators were guided by community grid members or village doctors during the household visits. Unified questionnaire forms and standardized survey terminologies were used to conduct face‐to‐face questionnaire surveys. Investigators were required to conduct one‐on‐one scale assessments with subjects to eliminate mutual interference. The investigation was divided into two stages: screening and diagnosis.

**Stage 1: Cognitive and Functional Screening**

The first stage involved the face‐to‐face investigation and neuropsychological assessment. A cognitive assessment was performed using the Chinese Mini‐Mental Status Examination (CMMS). A culturally adapted version of the Mini‐Mental State Examination (MMSE) is extensively utilized for cognitive screening among Chinese populations. This assessment evaluates several cognitive domains, including orientation, registration, attention and calculation, recall, and language. Following the cognitive impairment assessment guideline established by the Alzheimer's Disease Research Group of the Neurology Department of Peking Union Medical College Hospital and the China Cognitive and Aging Research Group of Xuanwu Hospital Capital Medical University: a participant was deemed to have cognitive function impairment based on the illiterate group ≤ 19 points, the primary school group ≤ 22 points, the junior high school and above group ≤ 26 points, and the result was recorded as positive; otherwise it was negative [22].

The Activity of Daily Living (ADL) was used to assess living ability. A subject was deemed to have impaired daily living ability based on the standard criteria of two or more items ≥ 3 points, or a total score ≥ 26, and the result was recorded as positive; otherwise, it was negative [23].

Participants who received negative results on both the CMMS and ADL assessments were classified as dementia‐negative (*n* = 579, 21.3%). Those with positive results on either assessment (*n* = 2133, 78.7%) proceeded to Stage 2 for diagnostic evaluation.

**Stage 2: Clinical Diagnosis**

Participants with negative CMMS and ADL scores were deemed negative for dementia. The subjects who passed this stage were enrolled in the diagnostic process stage, where they were screened using the “The Diagnostic and Statistical Manual of Mental Disorders, fourth edition, text revision (DSM‐IV‐TR)” by the American Psychiatric Association (APA) as the standard.

### 2.7 Ethics Statement

This study was approved by the Ethics Committee of the First People's Hospital of Zunyi City (No. (2022)‐1‐25), and screening was conducted with written informed consent from the subjects or guardians. All procedures performed in studies involving human participants were in accordance with the 1964 Helsinki declaration and its later amendments or comparable ethical standards.

### 2.8 Statistical Analysis

Statistical analysis was performed using SPSS 29.0 (IBM Corp., Armonk, NY, USA). Kolmogorov–Smirnov and Levene tests were used to perform normality and homogeneity of variance tests. For continuous variables that showed a normal distribution, they were presented as the mean ± standard deviation. Two groups were compared using the independent sample *t*‐test. Data that showed a severely skewed distribution were analyzed using the Mannu–Whitney *U* test. Measurement data were represented by *n* (%), and comparison between groups was performed by the Chi‐square test or Fisher exact probability method. The age and gender standardized prevalence rates were calculated using data from China's seventh national population census. The 95% confidence interval (CI) estimates for dementia prevalence rates were calculated using the ratios for the overall population and stratified by age, gender, and education level. Univariate logistic regression was performed to identify potential risk factors, which were then included in the multivariate logistic regression analysis and subjected to the stepwise input method for variables to determine the independent risk factors of dementia. *p* < 0.05 was considered statistically significant.

## 3 Results

### 3.1 General Information

**FIGURE 1:**
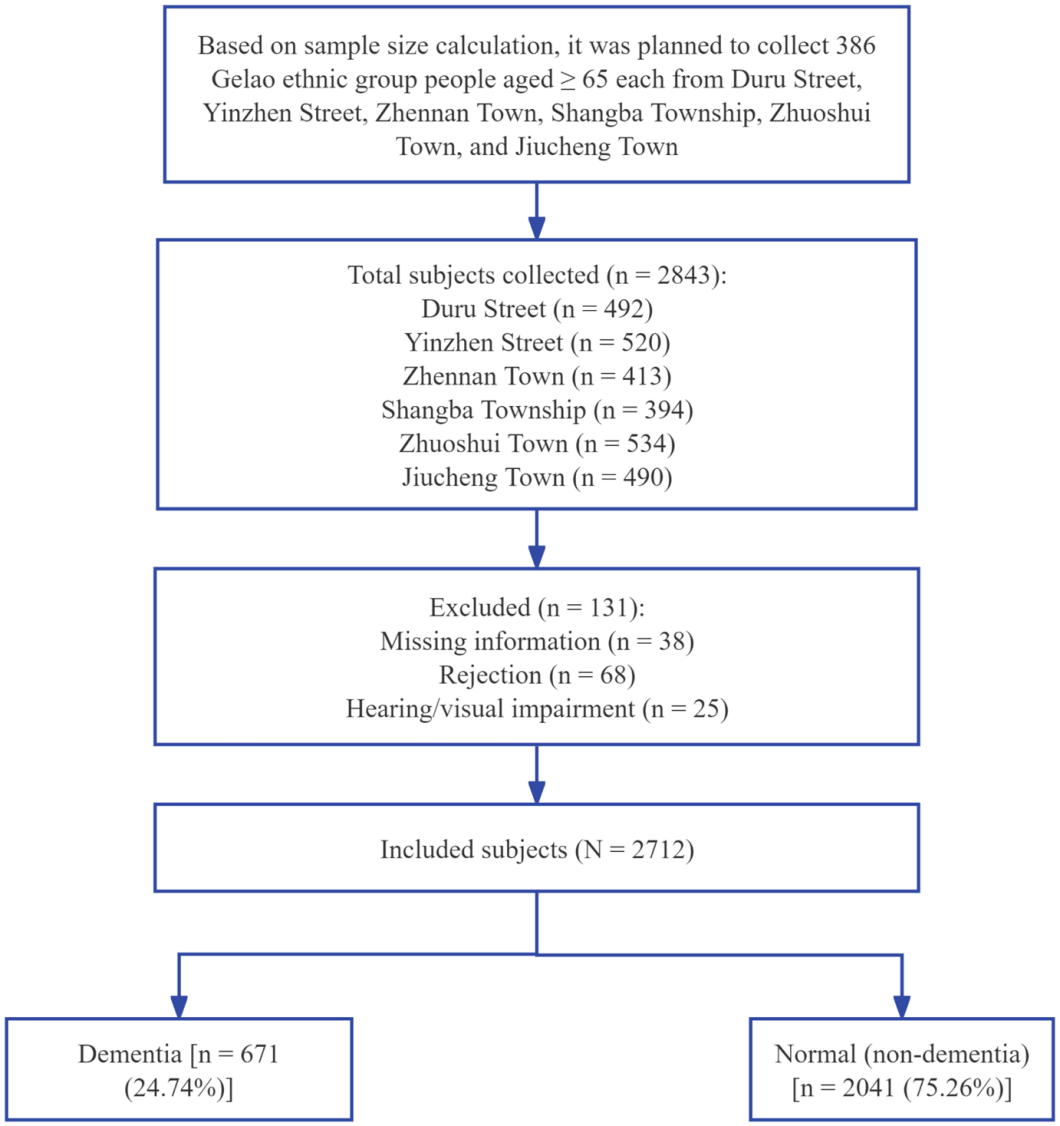
The flow chart of subject distribution and analysis.

A total of 2843 subjects were enrolled in this study, among whom 2712 provided valid survey questionnaires, representing an effective response rate of 95.39% (Figure 1). More detailed information is presented in Table 1.

**TABLE 1:** Distribution of the prevalence of dementia by age, gender, and education level.

Variables	Number of dementia cases %	95% CI	Number of males with dementia %	95% CI	Number of females with dementia%	95% CI
Total	671 (24.74)	23.10–26.37	237 (17.45)	15.43–19.47	434 (32.05)	29.56–34.54
Age						
65–69	87 (12.41)	9.96–14.86	31 (8.83)	5.86–11.80	56 (16.00)	12.16–19.84
70–74	158 (19.77)	17.01–22.53	45 (10.69)	7.75–13.63	113 (29.89)	25.27–34.51
75–79	167 (26.76)	23.29–30.23	54 (18.31)	13.94–22.68	113 (34.35)	29.21–39.49
80–84	147 (39.10)	34.16–44.04	64 (33.68)	26.94–40.42	83 (44.62)	37.46–51.78
85 years old and older	112 (52.83)	46.11–59.55	43 (42.57)	32.91–52.23	69 (62.16)	53.06–71.26
Education level						
Illiteracy	519 (30.35)	28.17–32.53	119 (22.28)	18.75–25.81	400 (34.01)	31.31–36.71
Primary school	95 (15.47)	12.61–18.33	66 (13.75)	10.67–16.83	29 (21.64)	14.66–28.62
Junior high school and above	57 (14.69)	11.16–18.22	52 (15.12)	11.34–18.90	5 (11.36)	1.99–20.73

*Note:* Data were presented as *N*, total number of included subjects, *n*, number of cases and (%) percentage.

### 3.2 The Overall Prevalence of Senile Dementia Among the Gelao Ethnic People

A total of 2712 subjects were included in the study, of which 671 met the diagnostic criteria for dementia. The overall prevalence rate of dementia was 24.74% (95% CI: 23.1–26.4). Based on the data from China's seventh national population census, the gender and age‐standardized rates were 23.88% and 22.81%, respectively. The analysis indicated a significant variation in the prevalence of dementia based on demographic characteristics. Notably, women exhibited a substantially higher prevalence than men (32.00% vs. 14.90%, *p* < 0.05), a trend that remained consistent across all age groups. The prevalence of dementia increased markedly with age, escalating from 12.40% in individuals aged 65–69 years to 52.8% in those aged ≥ 85 years. Furthermore, an inverse association was observed between education level and dementia prevalence, with the highest rate (30.40%) recorded among illiterate participants (Table 1, Figures S1 and S2).

### 3.3 Univariate Analysis of Basic Information on Dementia in Old Gelao Ethnic People

In terms of demographic data, the differences in the prevalence of dementia were statistically significant across different groups of gender, age, marital status, farmers, education level, living conditions, and average monthly family income (*p* < 0.05). There was no statistically significant difference in the occurrence of dementia between hand dominance (*p* > 0.05) (Table 2).

**TABLE 2:** Univariate analysis of the demographic data.

Variable	Total (*n* = 2712)	Non‐dementia (*n* = 2041, 75.3%)	Dementia (*n* = 671, 24.7%)	*β*	OR (95% CI)	*p*
Gender						
Male	1358 (50.10%)	1121 (54.90)	237 (35.30)	Reference		
Female	1354 (49.90%)	920 (45.10)	434 (64.70)	0.803	2.231 (1.862–2.673)	< 0.0001
Age (year)	74.3 ± 6.314	73.3 ± 5.832	77.37 ± 6.731	0.102	1.107 (1.091–1.123)	< 0.0001
65–69	701 (25.80%)	614 (30.10)	87 (13.00)	Reference		
70–74	799 (29.50%)	641 (31.40)	158 (23.50)	0.554	1.740 (1.309–2.311)	< 0.0001
75–80	624 (23.00%)	457 (22.40)	167 (24.90)	0.947	2.579 (1.937–3.433)	< 0.0001
80–84	376 (13.90%)	229 (11.20)	147 (21.90)	1.511	4.530 (3.338–6.149)	< 0.0001
≥ 85	212 (7.80%)	100 (4.90)	112 (16.70)	2.067	7.904 (5.565–11.227)	< 0.0001
Marital status						
Married	1804 (66.50%)	1396 (68.40)	408 (60.80)	Reference		
Single/divorced/widowed	908 (33.50%)	645 (31.60)	263 (39.20)	0.333	1.395 (1.164–1.672)	< 0.0001
Dominant hand						
Right hand	2682 (98.90%)	2023 (99.10)	659 (98.20)	Reference		
Left hand	30 (1.10%)	18 (0.90)	12 (1.80)	0.716	2.047 (0.981–4.271)	0.056
Farmer						
No	214 (7.90%)	188 (9.20)	26 (3.90)	Reference		
Yes	2498 (92.10%)	1853 (90.80)	645 (96.10)	0.923	2.517 (1.654–3.830)	< 0.0001
Education level						
Illiterate	1710 (63.05%)	1191 (58.40)	519 (77.30)	Reference		
Primary school	614 (22.64%)	519 (25.40)	95 (14.20)	−0.867	0.420 (0.330–0.535)	< 0.0001
Junior high school and above	388 (14.31%)	331 (16.20)	57 (8.50)	−0.928	0.395 (0.293–0.533)	< 0.0001
Residential status						
Nursing home	31 (1.10%)	17 (0.80)	14 (2.10)	Reference		
Living alone	317 (11.70%)	257 (12.60)	60 (8.90)	−1.261	0.283 (0.132–0.607)	0.001
Living with spouse	958 (35.30%)	724 (35.50)	234 (34.90)	−0.935	0.392 (0.191–0.808)	0.011
Living with children	739 (27.20%)	532 (26.10)	207 (30.80)	−0.750	0.472 (0.229–0.976)	0.043
Living with children and spouse	667 (24.60%)	511 (25.00)	156 (23.20)	−0.992	0.371 (0.179–0.769)	0.008
Average monthly household income, RMB						
< 1000	1344 (49.60%)	930 (45.60)	414 (61.70)	Reference		
1000–3000	1208 (44.50%)	974 (47.70)	234 (34.90)	−0.617	0.540 (0.449–0.649)	< 0.0001
≥ 3000	160 (5.90%)	137 (6.70)	23 (3.40)	−0.975	0.377 (0.239–0.595)	< 0.0001

*Note:* Data were presented as *N*, total number of included subjects; *n*, number of dementia cases and percentage (%).

### 3.4 Univariate Analysis of the Lifestyle and Dietary Habits Influencing the Risk of Dementia

For such diet and lifestyle habits as brushing teeth, participating in physical activities, going to the market, playing mahjong, smoking, drinking, drinking oil tea, eating porcini, and the frequency of spraying pesticides, the prevalence of dementia was significantly different (*p* < 0.05) (Table 3).

**TABLE 3:** Univariate analysis of lifestyle and dietary habits.

Variable	Total (*n* = 2712)	Non‐dementia (*n* = 2041, 75.3%)	Dementia (*n* = 671, 24.7%)	*β*	OR (95% CI)	*p*
Brushing teeth						
Never	319 (11.80%)	164 (8.00)	155 (23.10)	Reference		
2–5 times/week	108 (4.00%)	81 (4.00)	27 (4.00)	−1.042	0.353 (0.217–0.574)	< 0.0001
Once/day	1258 (46.40%)	965 (47.30)	293 (43.70)	−1.136	0.321 (0.249–0.415)	< 0.0001
2 times/day	799 (29.50%)	648 (31.70)	151 (22.50)	−1.400	0.247 (0.186–0.327)	< 0.0001
≥ 3 times/day	228 (8.40%)	183 (9.00)	45 (6.70)	−1.346	0.260 (0.176–0.385)	< 0.0001
Participate in physical activities						
No	117 (4.30%)	35 (1.70)	82 (12.20)	Reference		
Yes	2595 (95.70%)	2006 (98.30)	589 (87.80)	−2.077	0.125 (0.083–0.188)	< 0.0001
Going to the market						
No	1326 (48.90%)	914 (44.80)	412 (61.40)	Reference		
Yes	1386 (51.10%)	1127 (55.20)	259 (38.60)	−0.674	0.510 (0.427–0.609)	< 0.0001
Playing mahjong						
No	2238 (82.50%)	1604 (78.60)	634 (94.50)	Reference		
Yes	474 (17.50%)	437 (21.40)	37 (7.80)	−1.541	0.214 (0.151–0.303)	< 0.0001
Smoking						
No	1852 (68.30%)	1334 (65.40)	518 (77.20)	Reference		
Yes	860 (31.70%)	707 (34.60)	153 (22.80)	0.585	1.794 (1.466–2.196)	< 0.0001
Drinking alcohol						
No	2129 (78.50%)	1559 (76.40)	570 (84.90)	Reference		
Yes	583 (21.50%)	482 (23.60)	101 (15.10)	0.557	1.745 (1.379–2.207)	< 0.0001
Drinking *Camellia oleifera*						
No	1684 (62.10%)	1221 (59.80)	463 (69.00)	Reference		
Yes	1028 (37.90%)	820 (40.20)	208 (31.00)	−0.402	0.669 (0.555–0.806)	< 0.0001
Eating *Houttuynia cordata*						
No	760 (28.00%)	493 (24.20)	267 (39.80)	Reference		
Yes	1952 (72.00%)	1548 (75.80)	404 (60.20)	−0.730	0.482 (0.401–0.580)	< 0.0001
Eating *Boletus edulis*						
No	1038 (38.30%)	712 (34.90)	326 (48.60)	Reference		
Yes	1674 (61.70%)	1329 (65.10)	345 (51.40)	−0.567	0.567 (0.475–0.677)	< 0.0001
Pesticide use					0.567 (0.475–0.677)	
Never	897	641 (31.40)	256 (38.20)	Reference		
1–10 times/year	1614 (59.50%)	1246 (61.00)	368 (54.80)	−0.302	0.740 (0.614–0.891)	0.001
≥ 10 times/year	201 (7.40%)	154 (7.50)	47 (7.00)	−0.269	0.764 (0.535–1.092)	0.140

*Note:* Data were presented as *N*, total number of included subjects; *n*, number of cases and percentage (%).

### 3.5 Univariate Analysis of Health Status Influencing the Risk of Dementia in Old Gelao Ethnic People

In terms of health status, between various groups of sleep disorders, cerebrovascular diseases, headache history, and hypertension, the differences in the prevalence of dementia were statistically significant (*p* < 0.05). For obesity, early awakening, general anesthesia, brain trauma, CO poisoning, TIA, diabetes, and heart disease, the differences in the prevalence of dementia were not statistically significant (*p* > 0.05). The details are shown in Table S1.

### 3.6 Multivariate Analysis of Senile Dementia in the Gelao Ethnic Group

Multivariable logistic regression analysis revealed several factors that are independently associated with dementia. Older age (70–74 years: OR = 1.63, 95% CI: 1.20–2.22; 75–79 years: OR = 2.46, 95% CI: 1.79–3.38; 80–84 years: OR = 4.51, 95% CI: 3.18–6.40; ≥ 85 years: OR = 7.36, 95% CI: 4.86–11.14). Additional factors positively correlated with dementia include female gender (OR = 2.06, 95% CI: 1.60–2.65), low education level (comparing primary school to illiteracy: OR = 0.56, 95% CI: 0.41–0.75), occupation as a farmer (OR = 2.02, 95% CI: 1.23–3.34), a history of cerebrovascular disease (OR = 1.89, 95% CI: 1.43–2.49), and a history of hypertension (OR = 1.27, 95% CI: 1.03–1.57).

In contrast, factors negatively correlated with dementia include not residing in a nursing home (living alone vs. nursing home: OR = 0.20, 95% CI: 0.08–0.47), maintaining good tooth brushing habits (2 times/day vs. never: OR = 0.31, 95% CI: 0.22–0.43), engaging in physical activity (OR = 0.17, 95% CI: 0.11–0.28), visiting the market (OR = 0.17, 95% CI: 0.11–0.28), playing mahjong (OR = 0.37, 95% CI: 0.25–0.55), having a high income (≥ 3000 yuan vs. < 1000 yuan: OR = 0.57, 95% CI: 0.33–0.98), consuming oil tea (OR = 0.66, 95% CI: 0.53–0.83), and eating *Houttuynia cordata* roots (OR = 0.73, 95% CI: 0.59–0.90). All associations were statistically significant (*p* < 0.05), as shown in Table S2.

## 4 Discussion

In this study, we found that the prevalence of dementia among rural elderly people of Gelao ethnic above 65 years old was 24.74% (95% CI: 23.1–26.4). The prevalence among women was twice as high as that among men. In addition, the prevalence of dementia among those aged ≥ 85 was even higher, reaching 52.83%. Not living in a nursing home, having good tooth brushing habits, participating in physical activities, going to the market, playing mahjong, having a high‐income level, drinking *Camellia oleifera*, and eating *Houttuynia cordata* roots were associated with lower odds of dementia. Multivariate analysis showed that advanced age, female gender, low education level, occupation as farmers, a history of cerebrovascular disease, and a history of hypertension are associated with increased odds of dementia.

### 4.1 The Prevalence of Dementia Among the Gelao Ethnic People in China Was Significantly Higher Than the National Average

The analysis revealed that the prevalence of dementia in the Gelao ethnic population aged ≥ 65 was 24.74%, which was significantly higher than the national average [20], northern China [24], Chongqing Municipality [25], Zunyi City [26], as well as the prevalence of dementia among Han and other ethnic groups [27, 28, 29, 30, 31]. Previous studies demonstrated that a low education level increased the risk of developing dementia [31, 32, 33]. Consistently, this survey found that the illiteracy rate of elderly people of the Gelao ethnic group was 63.05%, but epidemiological studies in rural areas in northern China reported illiteracy rates of only 10.8% among the local Han population [34]. The cognitive flow study based on data from the longitudinal study of health and retirement in China revealed that the illiteracy rate among the old population was only 1.88% [35]. In the study, the proportion of illiterate people reached 63.05%, which might be a major factor driving the high prevalence of dementia. In addition, the study demonstrated that the proportion of people aged 70–84 in the region was higher than that of China's seventh national population census data [32]. This implies that the longer average life expectancy of elderly people in the region was another important factor contributing to the high prevalence of dementia. In the present study, the prevalence of dementia among the elderly women of Gelao ethnicity was significantly higher than that in men, and the prevalence of women in all age groups was almost twice that of men, which was significantly higher compared with the national average [30]. This is likely due to the underdevelopment level in the region. Due to long‐term historical reasons and customs, women have much lower opportunities for education than men, resulting in a much higher illiteracy rate among women than in men.

### 4.2 Special Food

Consumption of *Houttuynia cordata* roots and *Camellia oleifera* was associated with lower odds of dementia. Notably, the survey revealed that the *Houttuynia cordata* roots were an essential home vegetable in the region. Recent pharmacological research has demonstrated that *Houttuynia cordata* exhibits antibacterial and antiviral properties [36]. Recent studies have shown that *Houttuynia cordata* exhibits neuroprotective properties, including the inhibition of tau hyperphosphorylation and cholinergic dysfunction [37], and that sodium houttuyfonate, a component of this plant, can ameliorate memory impairment through anti‐inflammatory pathways [38].

*Camellia oleifera* is a popular tea consumed by the Gelao ethnic people, which is made by decocting lard or rapeseed oil, green tea, sesame, rice, soybeans, lard crackling, salt, etc. This ethnic group believes that *Camellia oleifera* can refresh the mind and improve constipation and indigestion [39]. Numerous studies have shown that tea polyphenols exhibit significant antioxidant properties, which may contribute to delaying brain aging and enhancing cognitive function [39, 40, 41, 42].

In addition, the elderly population of the Gelao ethnicity preferred to eat pickled cured meat, sausages, beef and mutton, etc. Previous investigations have shown that a large intake of red meat may elevate the concentration of low‐density lipoprotein and triglyceride in the blood circulation, promoting the formation of atherosclerotic plaque and increasing the risk of stroke [42, 43, 44]. Processed meat typically contains preservatives, most of which have large quantities of sodium and nitrite. High sodium levels can reduce arterial compliance, cause vascular stiffness, and elevate the risk of hypertension and stroke [45]. Meanwhile, sodium‐rich foods can cause cognitive decline and increase the risk of dementia in old people [46].

### 4.3 Social Activities

This survey found that elderly people often participated in mahjong and market activities, and the prevalence of dementia was significantly lower in those engaged in these activities than in those who did not. Participation in social activities has been reported to stimulate excitement in the central nervous system and promote the formation of neural networks in the brain, thereby enhancing adaptive changes in both brain structure and function [47]. The elderly people of the Gelao ethnicity often go to markets, which provides them with an avenue for sharing their emotions. Given the geographical limitations, the Gelao ethnic people have limited contact with each other in their daily life, and therefore, they use market gatherings as a platform for exchanging emotions. Moreover, it is particularly important to note that the Gelao ethnic people enjoy activities such as playing mahjong. A large prospective cohort study found that active participation in intellectual activities such as playing cards, mahjong, etc. helped to delay or prevent the occurrence of dementia among the elderly [47, 48, 49].

### 4.4 Brushing Teeth Condition

Our results indicated that old people who brushed their teeth once or less per day were 62.13%, and the prevalence of dementia was much higher in the non‐brushing population than in the brushing population. Similarly, researchers have reported that people who brush their teeth once or less a day are more likely to suffer from chronic diseases than those who brush their teeth twice or more a day [50]. A recent study demonstrated that the occurrence of *Porphyromonas gingivalis* due to non‐brushing teeth increased the risk of AD [51]. This bacterium disseminates from the oral cavity to the brain, releasing gingival protease that damages nerve cells, causing memory loss and cognitive impairment, and ultimately contributing to the development of AD [52]. A significant factor contributing to the high proportion of individuals who brush their teeth once or less per day is the lack of care services and educational programs aimed at improving the oral hygiene of the elderly in primary‐level hospitals and communities. This deficiency has led to an increase in cases of periodontitis, tooth loss, and other related conditions.

### 4.5 Economic Status and Physical Activities

This study identified a correlation between high income levels and reduced odds of dementia among elderly individuals in the Gelao ethnic group. A cohort study showed that the risk of developing dementia was 1.68 times greater in individuals belonging to the lowest personal wealth category [53]. The Gelao ethnic people in the survey areas lived in the hinterland of southwest China, and their economic level was relatively low. Most of them lived in rural areas, and the elderly were mainly farmers. They grew crops once a year due to low land fertility, resulting in low and unstable monthly income, making them cautious about their daily expenses. This survey also found that 92.1% of the Gelao ethnic people were farmers, while data from a nationwide dementia survey in China reported that only 58.2% of the population worked as farmers [54], indicating that the Gelao ethnic people are predominantly farmers. In addition, elderly individuals who regularly engaged in agricultural activities demonstrated significantly lower rates of dementia compared to those who did not. The Gelao ethnic group has resided in mountainous regions for decades, frequently moving uphill and downhill as part of their daily farming routines, which suggests they are subjected to high‐intensity physical activity. Numerous studies have demonstrated that consistent participation in physical activities can lower the risk of various chronic diseases, thereby mitigating cognitive decline and the onset of dementia in older adults [54, 55, 56, 57].

### 4.6 Residential Situation

Further analysis indicated that residing in a nursing home is associated with an elevated risk of developing dementia. In China, a significant number of elderly individuals reside in nursing homes due to factors such as insufficient family support, challenges in managing behavioral disorders, increased caregiving demands, and deteriorating health conditions [58]. Considering that those living in nursing homes are often alone, they are prone to develop frustration compared with those living at home. Elderly people might experience varying degrees of disability that restrict their participation in activities, leading to feelings of inferiority that further reduce their communication with the outside world. They might lose their social roles and feel even lonelier. Studies have demonstrated that the lonelier older individuals feel, the greater their cognitive deterioration [59].

### 4.7 Public Health Implications

The findings of this study carry significant implications for public health practice and policymaking. Notably, the prevalence of dementia within the elderly population of the Gelao ethnic group is significantly higher than the national average [20], which requires high attention from local and national health departments. This disparity indicates an urgent need for targeted dementia screening programs in ethnic minority regions, particularly in remote areas of Guizhou that have limited medical resources. Additionally, the protective factors identified in this study—including physical activity, social participation (such as visiting markets and playing mahjong), and specific dietary habits (including the consumption of oil tea and *houttuynia cordata* )—suggest potential avenues for community intervention programs. Health education and promotion initiatives can encourage the continuation of these culturally significant activities and dietary practices among the Gelao population. Furthermore, the independent risk factors identified (such as increasing age, female gender, low education level, agricultural occupation, and histories of cerebrovascular disease and hypertension) can assist healthcare professionals in identifying high‐risk groups for early screening and intervention. Since many of these risk factors are modifiable (such as hypertension and insufficient physical activity), incorporating dementia prevention strategies into existing chronic disease management programs for hypertension and cerebrovascular disease within primary healthcare settings may prove effective. Lastly, policymakers should consider allocating resources to develop dementia‐friendly communities in ethnic minority areas. This includes training village doctors for early dementia identification, establishing referral pathways to specialized medical institutions, and providing support services for caregivers. These tailored measures, aligned with the cultural and socioeconomic context of the Gelao people, may help mitigate the dementia burden on this vulnerable population.

### 4.8 Limitations

First, as a cross‐sectional study, these findings are based on observational data and cannot establish causality, which may restrict the analysis of risk factors. Second, the results obtained may only apply to the Gelao ethnic group, and therefore, further research is needed to determine whether the results can be extrapolated to other ethnic minorities or Han ethnic groups. Third, in this study, a large proportion of participants reported no hypertension, diabetes, or heart disease, which might not have been diagnosed and treated in time. Fourth, there may be some degree of bias in family history. Most of the elderly reported no family history because their brothers and sisters lived far apart or did not care enough about each other, and hence, they were unaware of each other's condition, or their parents had passed away several years ago and had been forgotten. In some cases, it was inconvenient for some participants to disclose their family information. Fifth, we did not perform a dose–response analysis because of the practical challenges associated with accurately measuring daily dietary intake in this elderly population. To validate these associations and determine if these dietary factors have a causal effect on dementia prevention, future prospective studies should include quantitative dietary assessments, biochemical validation, and longitudinal follow‐up.

## 5 Conclusions

This study shows that the prevalence of dementia in the Gelao ethnic group is significantly higher than the national average. Moreover, the prevalence of dementia increases with age and is disproportionately higher among women. Several factors are associated with lower odds of developing dementia, including not residing in nursing homes, maintaining good oral hygiene, engaging in physical activity, participating in market visits, playing mahjong, having a higher income, consuming Camellia oleifera oil tea, and incorporating *Houttuynia cordata* roots into the diet. Conversely, advanced age, female gender, low educational attainment, employment in farming, a history of cerebrovascular disease, and a history of hypertension were associated with increased odds of dementia. It is recommended that the Chinese government implement targeted measures to address these factors and reduce the burden of dementia among vulnerable populations.

## Author Contributions

**Pan Cai, Xiaoling Zhao, Dan Meng:** conceptualization, methodology, software. **Pan Cai, Xiaoling Zhao, Dan Meng, Xiaoli Yuan:** data curation, writing – original draft preparation. **Mei He, Yuhong Long, Yan Zhang, Xia Wu, Bin Zhao, Xiaoling Zhao:** investigation. **Dan Meng, Pan Cai, Yong Luo:** supervision. **Dan Meng:** software, validation. **Yong Luo, Jinghuan Gan:** writing – reviewing and editing.

## Funding

This work was supported by the Science and Technology Plan Project of Zunyi City (HZ (2024) No. 444), Study on Prevalence and Influencing Factors of Senile Dementia of Gelao nationality in China (no. Zunshi Kehe HZ Zi (2024) No. 6).

## Ethics Statement

This study was approved by the Ethics Committee of the First People's Hospital of Zunyi City (No. (2022)‐1‐25), and screening was conducted with written informed consent from the subjects or guardians. All procedures performed in studies involving human participants were in accordance with the 1964 Helsinki declaration and its later amendments or comparable ethical standards.

## Consent

Informed consent was obtained from all subjects involved in the study.

## Conflicts of Interest

The authors declare no conflicts of interest.

## Supporting information


**Figure S1:** Distribution of the prevalence of dementia by gender and age group.
**Figure S2:** Prevalence of dementia by gender and education level.
**Table S1:** Univariate analysis of disease history.
**Table S2:** Multivariate analysis of the influencing factors for dementia.

## Data Availability

The data that support the findings of this study are available from the corresponding author upon reasonable request.
