# Supplementary material for: Prevalence and Risk Factors of Dementia Among Older People of Gelao Ethnicity in China: A National Cross‐Sectional Study
Source: CNS Neurosci Ther. 2026 May 11;32(5):e70897. doi: 10.1002/cns.70897 (PMC13158701; doi:10.1002/cns.70897)
Supplement: Supplementary file 1 — Figure S1: Distribution of the prevalence of dementia by gender and age group. Figure S2: Prevalence of dementia by gender and education level. Table S1: Univariate analysis of disease history. Table S2: Multivariate analysis of the influencing factors for dementia. [file CNS-32-e70897-s001.docx]

**Supplementary materials**

**
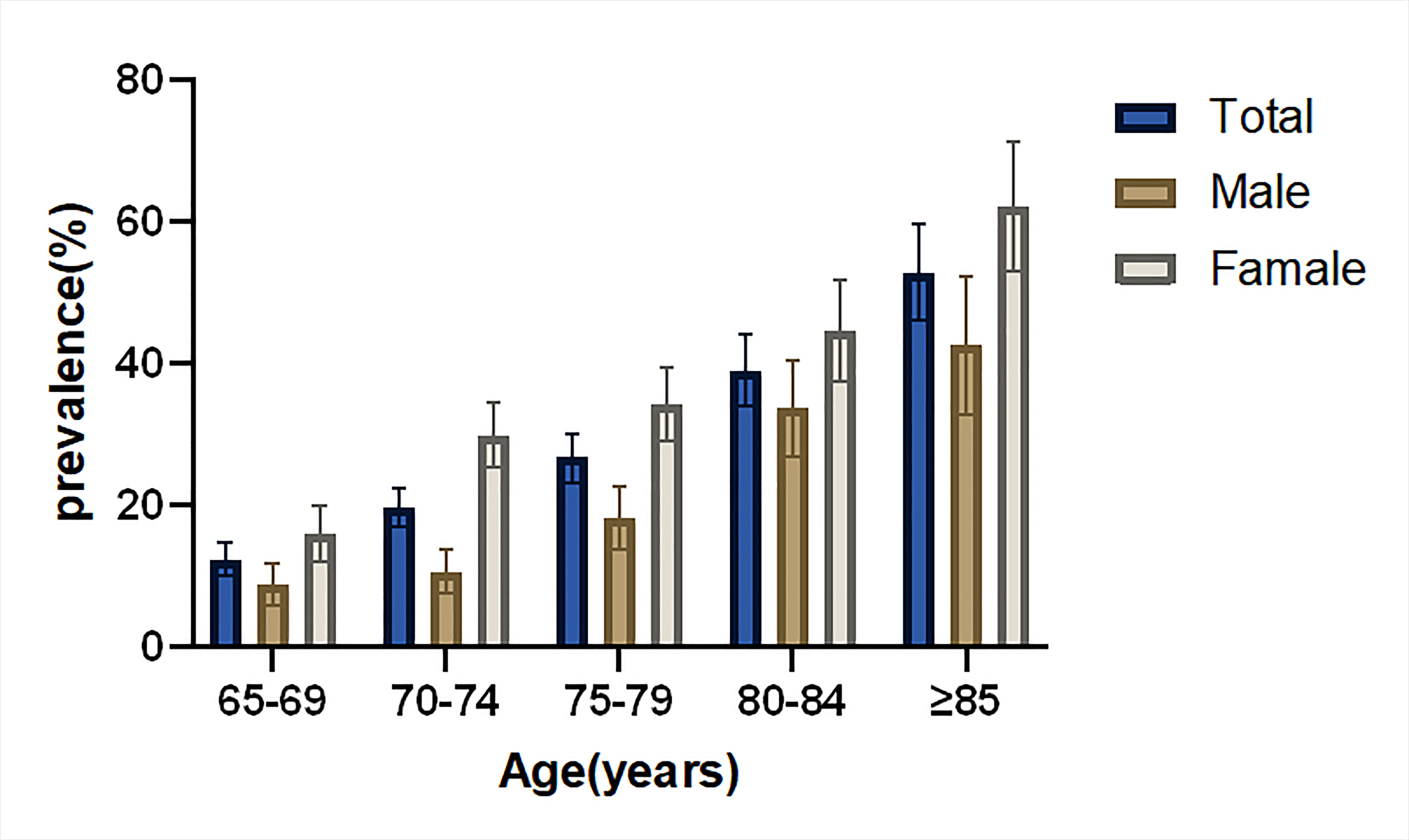
**

**Supplementary Figure 1**. Distribution of the prevalence of dementia by gender and age group.


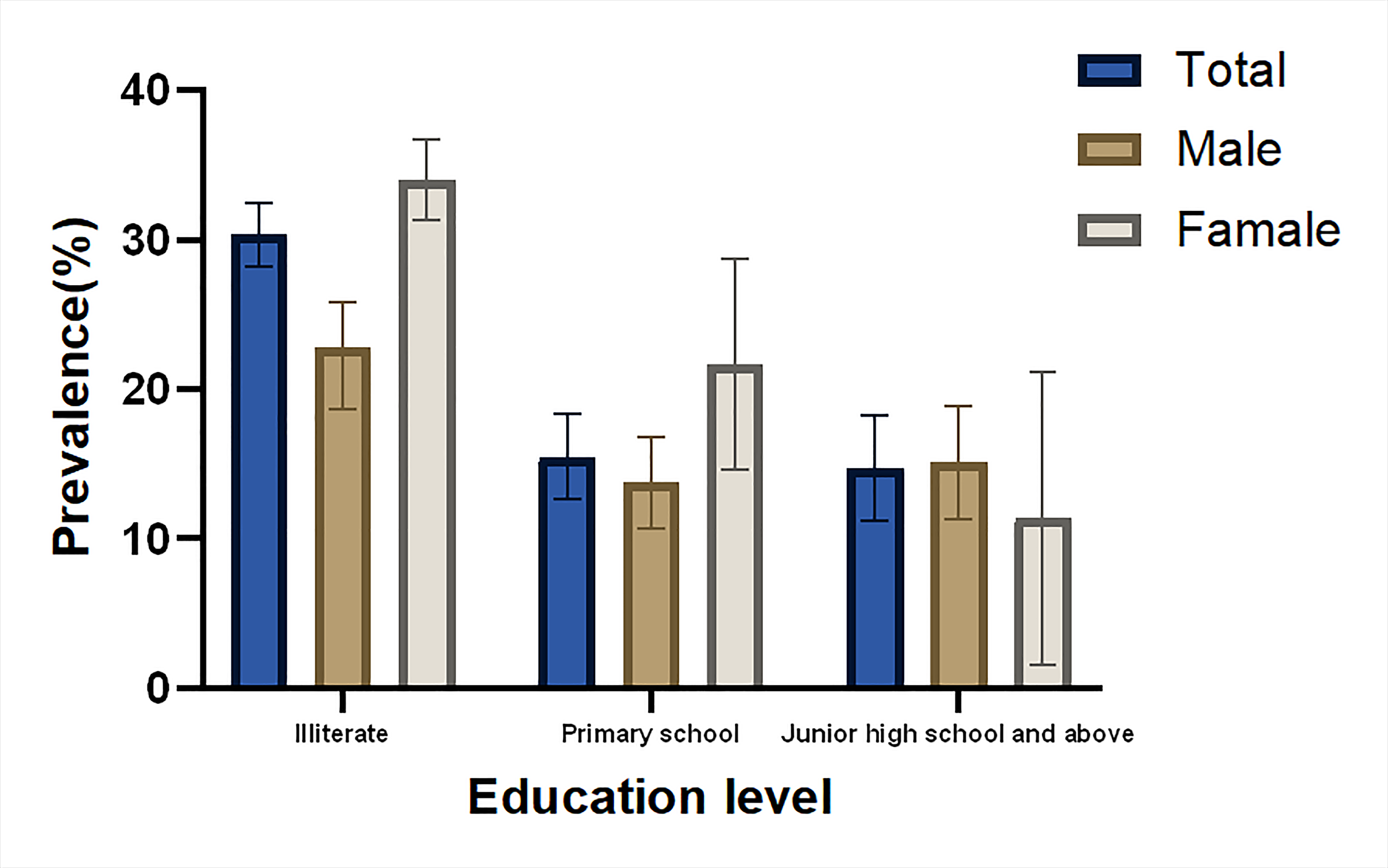


**Supplementary Figure 2**. Prevalence of dementia by gender and education level.

**Supplementary Table 1.** Univariate analysis of disease history.

| Variable | Total(n=2712) | Non-dementia (n = 2041, 75.3%) | Dementia  (n = 671, 24.7%) | β | OR (95%CI) | *P* |
| --- | --- | --- | --- | --- | --- | --- |
| **Obesity** |  |  |  |  |  |  |
| No | 2635 (97.20%) | 1986 (97.30) | 649 (96.70) | Reference |  |  |
| Yes | 77 (2.80%) | 55 (2.70) | 22 (3.30) | 0.202 | 1.224 (0.741-2.023) | 0.430 |
| **General anesthesia surgery** |  |  |  |  |  |  |
| No | 1910 (70.40%) | 1435 (70.30) | 475 (70.80) | Reference |  |  |
| Yes | 802 (29.60%) | 606 (29.70) | 196 (29.20) | -0.023 | 0.977 (0.807-1.183) | 0.813 |
| **Brain trauma** |  |  |  |  |  |  |
| No | 2469 (91.00%) | 1869 (91.60) | 600 (89.40) | Reference |  |  |
| Yes | 243 (9.00%) | 172 (8.40) | 71 (10.60) | 0.251 | 1.286 (0.961-1.721) | 0.091 |
| **CO poisoning** |  |  |  |  |  |  |
| No | 2697 (99.40%) | 2031 (99.50) | 666 (99.30) | Reference |  |  |
| Yes | 15 (0.60%) | 10 (0.50) | 5 (0.70) | 0.422 | 1.5252 (0.519-4.477) | 0.443 |
| **Cerebrovascular disease** |  |  |  |  |  |  |
| No | 2348 (86.60%) | 1812 (88.80) | 536 (79.90) | Reference |  |  |
| Yes | 364 (13.40%) | 229 (11.20) | 135 (20.10) | 0.690 | 1.993 (1.578-2.517) | <0.0001 |
| **TIA** |  |  |  |  |  |  |
| No | 2598 (95.80%) | 1957 (95.90) | 641 (95.50) | Reference |  |  |
| Yes | 114 (4.20%) | 84 (4.10) | 30 (4.50) | 0.87 | 1.090 (0.712-1.670) | 0.691 |
| **Headache** |  |  |  |  |  |  |
| No | 2294 (84.60%) | 1766 (86.50) | 528 (78.70) | Reference |  |  |
| Yes | 418 (15.40%) | 275 (13.50) | 143 (21.30) | 0.553 | 1.739 (1.390-2.176) | <0.0001 |
| **Diabetes** |  |  |  |  |  |  |
| No | 2573 (94.90%) | 1940 (95.10) | 633 (94.30) | Reference |  |  |
| Yes | 139 (5.10%) | 101 (4.90) | 38 (5.70) | 0.142 | 1.153 (0.786-1.692) | \| 0.466 \| \| --- \| |
| **Cardiovascular disease** |  |  |  |  |  |  |
| No | 2556(94.20%) | 1925(94.30) | 631(94.00) | Reference |  |  |
| Yes | 156(5.80%) | 116(5.70) | 40(6.00) | 0.51 | 1.052 (0.726-1.524) | 0.789 |
| **Hypertension** |  |  |  |  |  |  |
| No | 1732(63.90%) | 1349(66.10) | 383(57.10) | Reference |  |  |
| Yes | 980(36.10%) | 692(33.90) | 288(42.90) | 0.382 | 1.466 (1.227-1.752) | <0.0001 |

Note: Data were presented as N, total number of included subjects; n, number of cases and percentage (%).

**Supplementary Table 2.** Multivariate analysis of the influencing factors for dementia.

| **Variable** | **β** | **SE** | **Wald χ2** | **P** | **OR (95% *CI*)** |
| --- | --- | --- | --- | --- | --- |
| **Gender** |  |  |  |  |  |
| Male | 1.000 |  |  |  |  |
| Female | 0.723 | 0.128 | 31.826 | <0.0001 | 2.061(1.603-2.649) |
| **Age** |  |  |  |  |  |
| 65- 69 | 1.000 |  |  |  |  |
| 70-74 | 0.490 | 0.158 | 9.658 | 0.002 | 1.633(1.198-2.224) |
| 75- 79 | 0.901 | 0.162 | 30.787 | <0.0001 | 2.461(1.790-3.383) |
| 80-84 | 1.506 | 0.178 | 71.363 | <0.0001 | 4.510(3.180-6.396) |
| ≥85 | 1.996 | 0.211 | 89.143 | <0.0001 | 7.360(4.863-11.138) |
| **Education level** |  |  |  |  |  |
| Illiterate | 1.000 |  |  |  |  |
| Primary school | -0.589 | 0.153 | 14.804 | <0.0001 | 0.555(0.411-0.749) |
| Junior high school and above | -0.070 | 0.201 | 0.123 | 0.726 | 0.932(0.629-1.381) |
| **Residential status** |  |  |  |  |  |
| Nursing home | 1.000 |  |  |  |  |
| Living with spouse | -0.779 | 0.421 | 3.433 | 0.064 | 0.459(0.201-1.046) |
| Living with children | -1.193 | 0.424 | 7.912 | 0.005 | 0.303(0.132-0.696) |
| Living with children and spouse | -0.732 | 0.426 | 2.952 | 0.086 | 0.481(0.209-1.108) |
| Living alone | -1.624 | 0.442 | 13.519 | <0.0001 | 0.197(0.083-0.468) |
| **Brushing teeth condition** |  |  |  |  |  |
| Never | 1.000 |  |  |  |  |
| 2-5 times/week | -0.811 | 0.282 | 8.243 | 0.004 | 0.444(0.256-0.773) |
| 1 time/day | -0.825 | 0.155 | 28.336 | <0.0001 | 0.438(0.323-0.594) |
| 2 times/day | -1.176 | 0.172 | 46.801 | <0.0001 | 0.308(0.220-0.432) |
| 3 times/day | -1.156 | 0.232 | 24.714 | <0.0001 | 0.315(0.200-0.497) |
| **Participate in physical activities** |  |  |  |  |  |
| No | 1.000 |  |  |  |  |
| Yes | -1.765 | 0.243 | 52.977 | <0.0001 | 0.171(0.106-0.275) |
| **Farmer** |  |  |  |  |  |
| No | 1.000 |  |  |  |  |
| Yes | 0.705 | 0.255 | 7.620 | 0.006 | 2.023(1.227-3.337) |
| **Going to the market** |  |  |  |  |  |
| No | 1.000 |  |  |  |  |
| Yes | -0.567 | 0.107 | 28.084 | <0.0001 | 0.171(0.106-0.275) |
| **Playing mahjong** |  |  |  |  |  |
| No | 1.000 |  |  |  |  |
| Yes | -0.986 | 0.198 | 24.828 | <0.0001 | 0.373(0.253-0.550) |
| **Average monthly household income** |  |  |  |  |  |
| <1000 yuan | 1.000 |  |  |  |  |
| 1000-3000 yuan | -0.576 | 0.113 | 26.029 | <0.0001 | 0.562(0.451-0.702) |
| ≥3000yuan | -0.570 | 0.281 | 4.131 | 0.042 | 0.565(0.326-0.980) |
| **Eating *Houttuynia cordata* roots** |  |  |  |  |  |
| No | 1.000 |  |  |  |  |
| Yes | -0.319 | 0.111 | 8.327 | 0.004 | 0.727(0.585-0.903) |
| **Drinking *Camellia oleifera*** |  |  |  |  |  |
| No | 1.000 |  |  |  |  |
| Yes | -0.412 | 0.114 | 13.177 | <0.0001 | 0.662(0.530-0.827) |
| **Cerebrovascular disease** |  |  |  |  |  |
| No | 1.000 |  |  |  |  |
| Yes | 0.634 | 0.142 | 19.822 | <0.0001 | 1.886(1.426-2.493) |
| **Hypertension** |  |  |  |  |  |
| No | 1.000 |  |  |  |  |
| Yes | 0.240 | 0.107 | 4.976 | <0.0001 | 1.271(1.030-1.569) |
